# Posterior pole involvement as the presenting feature of varicella zoster virus- associated acute retinal necrosis in a young healthy man

**DOI:** 10.1186/s12348-025-00540-3

**Published:** 2025-11-21

**Authors:** Ravid Ben-Avi, Radgonde Amer

**Affiliations:** 1 Department of Ophthalmology, Hadassah Medical Center, Kiryat Hadassah, POB 12000, Jerusalem, 91120 Israel; 2 Faculty of Medicine, Hebrew University of Jerusalem, Jerusalem, Israel

**Keywords:** Acute retinal necrosis, Macular involvement, Posterior pole involvement, VZV retinitis

## Abstract

**Purpose:**

To report on the long-term clinical course of a young, healthy patient who presented with a solitary posterior pole lesion as the initial manifestation of acute retinal necrosis (ARN).

**Methods:**

Descriptive case report.

**Results:**

A 37-year-old man presented with a two-day history of right eye redness, pain and central scotoma. Examination revealed panuveitis and a solitary whitish-yellow retinal infiltrate in the posterior pole, with an otherwise normal retinal periphery. Given the clinical suspicion of Bartonella-associated retinitis, empiric systemic antibiotic therapy was initiated. One week later, new peripheral whitish-yellow retinal infiltrates emerged. Anterior chamber tap was positive for varicella zoster virus (VZV). The patient was diagnosed with acute retinal necrosis (ARN). Systemic and intravitreal antiviral therapy was initiated in combination with prednisone. All retinal lesions regressed completely. Areas of macular and peripheral retinal atrophy subsequently developed. At one-year of follow-up, visual acuity improved significantly from logMAR 1.0 to 0, with no disease recurrence or additional complications.

**Conclusions:**

Posterior pole involvement as the initial manifestation of ARN in immunocompetent individuals is uncommon. This case highlights the importance of recognizing atypical clinical phenotypes of ARN. While the intensive medical therapy did not prevent the loss of retinal tissue, it successfully halted the progression of the infection toward the fovea, thereby allowing the preservation of excellent final visual acuity. Although uncommon, macular involvement should be included in the differential diagnosis of viral retinopathies. Prompt diagnosis and appropriate antiviral therapy are critical to preserving visual function and preventing further complications.

## Introduction

Acute retinal necrosis (ARN) is a rare, sight-threatening condition characterized by acute panuveitis, retinal periarteritis, and progressive full-thickness necrotizing retinitis, which may result in retinal detachment (RD**)** [1]. Following resolution, the affected areas typically develop atrophic and gliotic scarring [2]. VZV is the most common cause, followed by HSV types 1 and 2 [1]. The estimated annual incidence is 0.5–0.63 cases per million in the UK [1].

In 1994, the Executive Committee of the American Uveitis Society established diagnostic criteria for ARN based on specific clinical features [3]: focal, well-demarcated areas of retinal necrosis located in the peripheral retina (outside of the major temporal vascular arcades); rapid, circumferential progression of necrosis (if antiviral therapy has not been administered); evidence of occlusive vasculopathy, and; a prominent inflammatory reaction in the vitreous and anterior chamber.

Retinal necrosis predominantly affects the peripheral retina in its early stages, with involvement of the macula generally arising only in the later phases of the disease, if it develops at all [4]. In this report, we describe the case of a healthy young man whose initial manifestation of ARN was a posterior pole retinal lesion.

## Case report

**Fig. 1:**
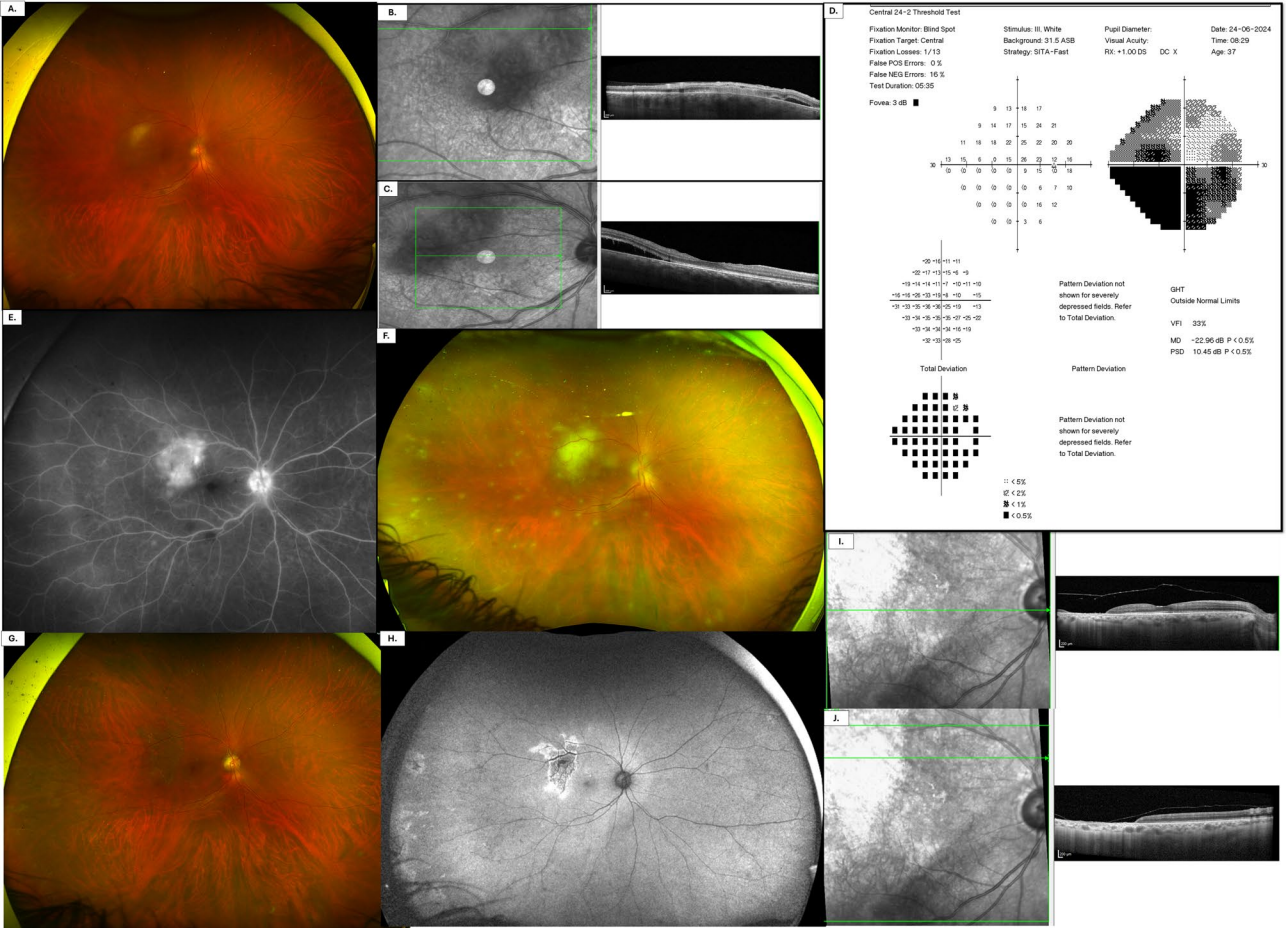
**A** Pseudo-color ultra-widefield fundus image of the right eye showing a whitish-yellow lesion located inferior to the superotemporal vascular arcade. **B–C** Spectral domain-optical coherence tomography scans of the right eye centered on the lesion show hyperreflectivity of outer retinal layers and minimal intraretinal fluid. Subretinal fluid is noted inferior to the retinal lesion (**D**) Visual field testing (SITA-Fast 24 − 2) of the right eye demonstrated a dense inferior scotoma, most pronounced in the inferonasal quadrant, corresponding to the location of the superior temporal retinal lesion. A mild defect was also noted in the superonasal visual field. **E** Ultra-widefield fluorescein angiography (FA) of the right eye shows late leakage of the retinal lesion and periphery with a hot optic disc (**F**) Pseudo-color ultra-widefield fundus images of the right eye seven days after the initial presentation, reveal new whitish-yellow peripheral retinal lesions accompanied by perivascular sheathing of the peripheral retinal vessels (**G**) After 11 months: Pseudo-color ultra-widefield fundus image of the right eye demonstrating resolution of both macular and peripheral retinal infiltrates. **H** Fundus autofluorescence (FAF) image showing central hypoautofluorescence corresponding to areas of macular and peripheral previous retinal lesions, with surrounding hyperautofluorescence at the lesion margins. **I-J** OCT image showing complete retinal atrophy in the area corresponding to the previously observed posterior pole lesion

A 37-year-old man presented with complaints of redness and pain in the right eye (RE) of two-day-duration, accompanied by the sudden onset of a central dark spot in his visual field. The patient was otherwise healthy, with no relevant systemic or ocular history. A review of systems was unremarkable. Ocular examination revealed in RE, a best corrected visual acuity (BCVA) of 6/7.5 and 6/6 in the left eye (LE). Intraocular pressure (IOP) measured 29 mmHg in RE and 22 mmHg in LE. Anterior segment examination of RE demonstrated conjunctival hyperemia with perilimbal injection, mutton-fat keratic precipitates (KPs), and anterior chamber cells graded + 3 with associated + 2 flare. Posterior segment examination showed a clear vitreous with a few inferior vitreous strands. The optic nerve head exhibited mildly blurred margins. Retina evaluation revealed a solitary whitish-yellow lesion located inferior to the supero-temporal vascular arcade. Within this lesion, sheathed intraretinal vessels were noted. The peripheral retina appeared normal (Fig. 1A). Examination of the left eye was unremarkable.

Spectral-domain optical coherence tomography (SD-OCT; Heidelberg Spectralis, Heidelberg, Germany) of RE retinal lesion revealed outer retinal hyperreflectivity with minimal intraretinal fluid. Subretinal fluid was predominantly noted adjacent to and inferior to the retinal lesion (Fig. 1B-C). Visual field examination (Sita-Fast 24 − 2) showed a dense inferior scotoma, most prominent on the inferior nasal quadrant, corresponding to the superior temporal lesion, with a milder defect on the superior nasal visual field. (Figure 1D**)**. Ultra-widefield fluorescein angiography (FA) demonstrated early blocked fluorescence and late leakage in the area of the retinal lesion in addition to late diffuse peripheral leakage (Fig. 1E). The left eye showed no abnormalities.

Based on the above findings, a diagnosis of right eye panuveitis was established, and an infectious etiology was suspected. The patient mentioned exposure to cats and this led to a presumptive diagnosis of bartonella infection. Empiric antibiotic therapy was initiated with rifampicin (300 mg twice per day) and doxycycline (100 mg twice per day). This was supplemented with topical corticosteroids, cycloplegic drops, and intraocular pressure-lowering medication. Routine blood tests including complete blood count, liver and kidney function tests and ESR were normal. Serological tests for bartonella, toxoplasma, syphilis and HIV were negative. Chest x-ray was normal. CD4 count was normal (134.4 × 10⁸/L).

Six days after the initial presentation, the retinal lesion looked bigger and new round, whitish-yellow peripheral retinal lesions developed, along with perivascular sheathing involving the peripheral retinal vessels. Because of suspected viral etiology, an anterior chamber tap (AC tap) was performed. Polymerase chain reaction (PCR) of aqueous humor revealed the presence of VZV DNA, and this was confirmed with a separate aqueous humor sample. The patient reported no concurrent skin rash or recent systemic illness. He recalled a history of chickenpox in childhood. A diagnosis of VZV-associated acute retinal necrosis (ARN) was established.

Antiviral treatment was initiated with intravenous acyclovir (10 mg/kg three times daily) for 2 weeks, in combination with intravitreal injections of foscarnet (2.4 mg/0.1 mL) and ganciclovir (2 mg/0.05 mL) administered on alternate days, totaling eight injections. Prednisone (1 mg/kg/day) was added 2 days later and it was tapered gradually. Extensive peripheral retinal involvement became evident in the following days, in addition to diffuse periphlebitis and occlusive arteriolitis (Fig. 1F).

Thereafter, the patient demonstrated gradual clinical improvement with resolution of the retinal infiltrates and absorption of subretinal fluid (Fig. 1G-H). Oral valacyclovir (2 g three times daily) was continued for a total of 3 months, and it was subsequently tapered to 1 g three times daily for another 3 months. The patient thereafter was kept on maintenance dose of acyclovir (800 mg twice daily) and prednisone 5 mg daily. BCVA one year later improved to 6/6. However, retinal atrophy developed in the area of the retinal infiltrate, which manifested as total loss of retinal tissue on SD-OCT (Fig. 1I-J). Given the risk of involvement in the contralateral eye, the patient was maintained on long-term antiviral prophylaxis.

## Discussion

This article reports on the long-term clinical course of an immunocompetent young man whose first presentation with ARN took the form of a solitary retinal infiltrate. This presentation is highly unusual, as ARN typically starts in the peripheral retina with later or absent macular involvement. We aim to highlight the favorable outcome despite early posterior pole involvement threatening the fovea. While the intensive systemic and intravitreal antiviral therapy did not prevent the loss of retinal tissue in the area of the retinal lesion in the posterior pole, it successfully halted the progression of the infection toward the fovea, thereby allowing the preservation of excellent final visual acuity.

The diagnostic course illustrates the challenges presented by atypical posterior pole lesions. Initially, the patient was suspected to have bartonella-associated retinitis based on his exposure history and solitary lesion, and was treated empirically with systemic antibiotics. However, the rapid progression of retinal necrosis, confirmed by PCR detection of VZV DNA in the aqueous humor, established a diagnosis of ARN. This case emphasizes the high diagnostic value of anterior chamber paracentesis in atypical presentations, even in otherwise healthy individuals.

The therapeutic rationale for our intensive regimen was based on both the lesion’s location adjacent to the fovea and the rapid peripheral extension observed. We administered systemic IV acyclovir followed by high-dose oral valacyclovir, in combination with alternating intravitreal injections of foscarnet and ganciclovir, to achieve both systemic and local viral suppression. Corticosteroids were introduced subsequently to control inflammation and limit immune-mediated damage. The optimal treatment protocol for ARN remains debated, particularly regarding the role of adjunctive intravitreal therapy and the appropriate duration of intravitreal foscarnet or ganciclovir administration. A 2016 report by the American Academy of Ophthalmology (AAO) [1], based on a comprehensive literature review, concluded that the most frequently reported initial treatments include intravenous acyclovir or oral valacyclovir, with evidence supporting both approaches for induction therapy as well as to reduce the risk of contralateral eye involvement. After induction therapy for 7–10 days, longer-term maintenance therapy (typically 1000 mg valacyclovir daily) for 6 months or more is common. The early use of intravitreal foscarnet al.ongside systemic acyclovir as adjunctive therapy for ARN is supported by the AAO report, which stated it may provide immediate therapeutic drug levels within the vitreous, help suppress viral replication, reduce the likelihood of severe vision loss and retinal detachment, and potentially offer improved effectiveness against herpes virus strains resistant to acyclovir [1]. In our case, although retinal tissue loss developed at the site of the infiltrate, this aggressive strategy likely played a critical role in halting progression toward the fovea and preserving central vision.

Importantly, although SD-OCT demonstrated total retinal atrophy at the lesion site, the fovea itself remained anatomically intact. This explains the excellent final BCVA of 6/6 achieved one year later despite the proximity of the necrotic lesion. The preservation of foveal architecture, even in the setting of adjacent retinal loss, highlights the potential benefit of early aggressive therapy in selected cases.

A review of the literature reveals that posterior pole involvement in ARN is uncommon, with only a limited number of cases documented to date (Table 1) and presented as follows.

**Table 1:** Summary of reported cases of acute retinal necrosis that primarily involved the posterior pole

Num. of cases	Age (years)/Gender	Systemic Status	Eye Involved	Virology (PCR result)	Concurrent Systemic Illness	Main Complaint	Posterior Pole Involvement At Presentation	VA at Presentation	VA at Last Follow Up	Complications	Additional Intervention
1	44/F	Healthy	LE	-	-	Blurred vision, redness and pain.	Multiple white isolated lesions, retinal vasculitis, perivenous hemorrhages, blurred disc margins.	20/25	4 weeks − 20/60 2 months (following RD) – CF	RRD (after 2 months)	RD repair surgery
Retrospective study, 12 eyes	57.4 ± 16.7 M: F ratio 1:2	Healthy	-	3 eyes positive for VZV	-	-	Posterior pole lesions – 12/12 Macular lesions − 6/12 Vascular sheathing − 7/12 Retinal Hemorrhages − 7/12 Optic disc edema – 6/12 Macular edema – 4/12	Mean − 20/227	Mean 2 years: 20/449	ERM − 7/12 RD − 10/12 Recurrence – 1/12	RD repair surgery in 7 eyes, 3 eyes with RD were not repaired. 1 eye required multiple procedures
1	50/F	Healthy	RE	VZV	-	Blurred vision	Macular swelling with perifoveal yellowish–white exudates and white patches of the peripheral retina.	6/20	1 month − 8/20	Macular atrophy	-
1	32/M	Healthy	RE	VZV	-	Decreased vision	Slight macular swelling with yellowish-white exudation	20/30	20/25	-	Prophylactic laser photocoagulation and PPV + SB due to disease progression despite therapy
1 (2 eyes)	34/F	Healthy	BE	-	Fever, vesicular rash, headache, vertigo, neck stiffness and joint pains.	Decreased vision	Area of retinal necrosis, retinal edema in posterior pole involving macula with scattered superficial retinal hemorrhages and cotton-wool spots	BE - CF	10 months: RE -CF 1 M; LE 6/18	-	-
1	40/F	Healthy	LE	HSV-1	-	Ocular pain during eye movements.	Dense VH, multiple subretinal white lesions around the optic disc, and retinal arterial sheathing. US - vitreous haziness, retinal and choroid edema, scleral thickening. OCT – SRF.	20/2000	1 month (following RD surgery) – HM	RRD (after 1 month)	RD repair surgery
2	24/M	Healthy	LE	HSV-1	-	Blurred vision and redness	Optic disc edema, retinal venous tortuosity	20/100	3 weeks − 20/66	-	-
3	41/M	Healthy	BE	HSV-1	Fever	RE - Sudden visual loss; LE - Blurred vision	RE - Massive retinal edema and scattered retinal hemorrhage; LE - Scattered retinal hemorrhage along vessels and an arterial sheath.	RE – NLP LE - HM	-	RE CRVO, CRAO and RD	-
1	31/F	Healthy	RE	VZV	-	Blurred vision, photophobia	Hyperemia of the optic disc, a discrete white-yellow exudate involving the fovea	20/100	10 months – 20/20	-	Valacyclovir 1000 mg daily for recurrence prevention
1	57/M	Healthy	BE	VZV	-	Blurred vision	Blurred optic disc margins, scattered hemorrhages in the posterior pole, and yellowish-white lesions at the macula	RE - CF LE - HM	20 days – RE – CF; LE NLP	-	--

*F* female, *M* male, *RD/RRD* rhegmatogenous retinal detachment, *CF* counting finger, *HM* hand motion, *NLP* No light perception, *PPV* pars plana vitrectomy, *SB* scleral buckle, *LE* left eye, *RE* right eye, *BE* both eyes, *ERM* epiretinal membrane, *VH* vitreous hemorrhage, *US* ultrasound, *OCT* optical coherence tomography, *SRF* subretinal fluid, *CRVO* central retinal vein occlusion, *CRAO* central retinal artery occlusion, *HSV* herpes simplex virus, *VZV* varicella zoster virus

A review of cases reported by Cartwright [5], Murata [6], Minamoto [7], Majumder [8], Hu [9], Wang [10] and Zhu [11]—excluding Margolis et al. [12] due to its retrospective case-series design—reveals a total of 12 eyes from 9 patients presenting with posterior pole involvement as the initial manifestation of ARN. Of these patients, 66% (6/9) had unilateral involvement, while 33% (3/9) exhibited bilateral disease. All individuals were described as immunocompetent. The mean age at presentation was 39 years (± 9.6), with a slight predominance of females (5 females, 4 males). PCR results were available for 7 patients: 4 tested positive for VZV and 3 for HSV-1. Only 2 of the 9 patients reported preceding systemic symptoms—one of whom experienced fever, while the other reported fever, joint pain, and a vesicular rash. Blurred vision was the most common presenting complaint, reported in 8 out of 9 patients. Posterior pole findings at presentation primarily included yellow-white macular exudates, optic disc edema, retinal hemorrhages, macular edema, and vascular sheathing. Retinal detachment was observed in 3 of the 12 affected eyes (25%). Each of the three patients who had retinal detachment (RD) were reported to have peripheral necrotic lesions [5, 9]. In comparison, Margolis et al. [12] described 12 patients with posterior pole involvement. These patients were older, with a mean age of 57.4 years (± 16.7), and the majority were females. Three patients were tested and each had positive PCR results for VZV. A significantly higher rate of retinal detachment was reported in this cohort—10 out of 12 eyes (83%). A review of these data underscores that ARN should be considered in young, otherwise healthy patients who present with blurred vision and posterior pole lesions, irrespective of accompanying systemic symptoms.

The pattern of posterior retinitis observed in our study together with the cases presented above exhibit significant clinical resemblance to progressive outer retinal necrosis (PORN), a distinct and severe form of necrotizing herpetic retinopathy. PORN is characterized by multifocal, well-demarcated white lesions localized to the posterior pole, which rapidly progress and coalesce. It predominantly affects individuals with profound immunosuppression and is typically marked by minimal or absent intraocular inflammation [12]. Margolis et al., along with Majumder et al. [8] suggested that patients with posterior pattern of retinitis appeared to have an intermediary form of necrotizing retinopathy, sharing features with both ARN and PORN. The former introduced a new acronym—multifocal posterior necrotizing retinitis (MPNR)—to describe this acute, progressive, inflammatory herpetic retinopathy characterized by multifocal necrosis involving the posterior pole, accompanied by vasculitis, vitritis, and anterior uveitis.

This case underscores the clinical significance of this rare presentation of posterior pole involvement as first manifestation of ARN. It emphasizes the need for a high index of suspicion for viral retinitis, even in immunocompetent patients. Additionally, we advocate for prompt anterior chamber paracentesis in such atypical cases, given its high diagnostic value for viral retinitis and its feasibility as an outpatient procedure. Moreover, intensive antiviral therapy can preserve excellent long-term vision, even when the macula is threatened at presentation.

## Conclusions

We describe a rare presentation of ARN in a young, otherwise healthy male patient whose initial manifestation involved the posterior pole. The patient underwent prompt, intensive therapy and achieved a favorable visual outcome. A review of the literature reveals only a handful of cases in which posterior pole lesions manifested as the initial sign of ARN. Although uncommon, macular involvement should be included in the differential diagnosis of viral retinopathies. This report contributes to the limited evidence on this atypical phenotype by detailing our treatment strategy and clinical results.

## Data Availability

No datasets were generated or analysed during the current study.
